# Supplementary figures and images for: Heterogenous Susceptibility to R-Pyocins in Populations of Pseudomonas aeruginosa Sourced from Cystic Fibrosis Lungs
Source: mBio. 2021 May 4;12(3):e00458-21. doi: 10.1128/mBio.00458-21 (PMC8262887; doi:10.1128/mBio.00458-21)

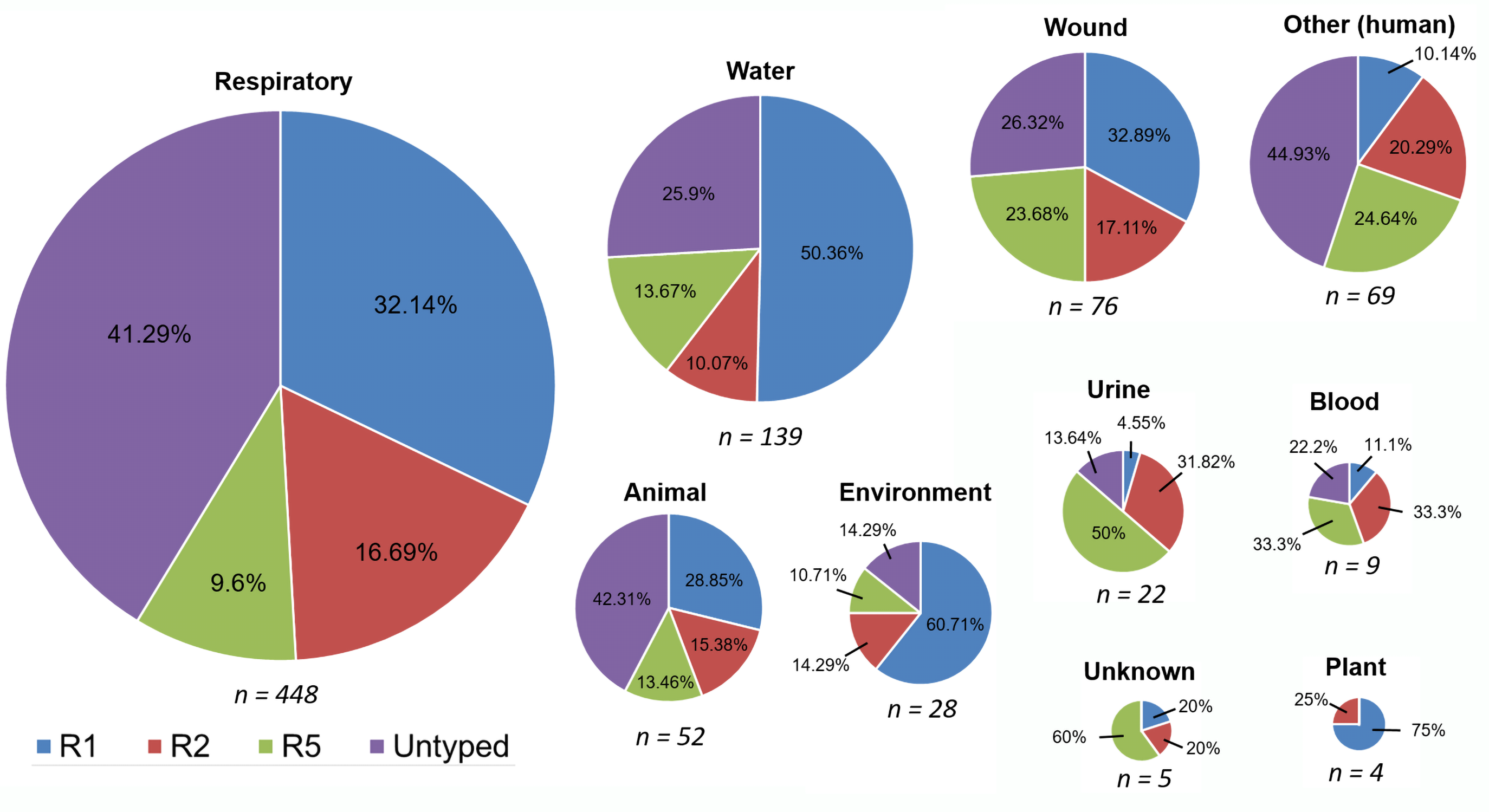

Supplement: FIG S1 [file mbio.00458-21-sf001.tif]

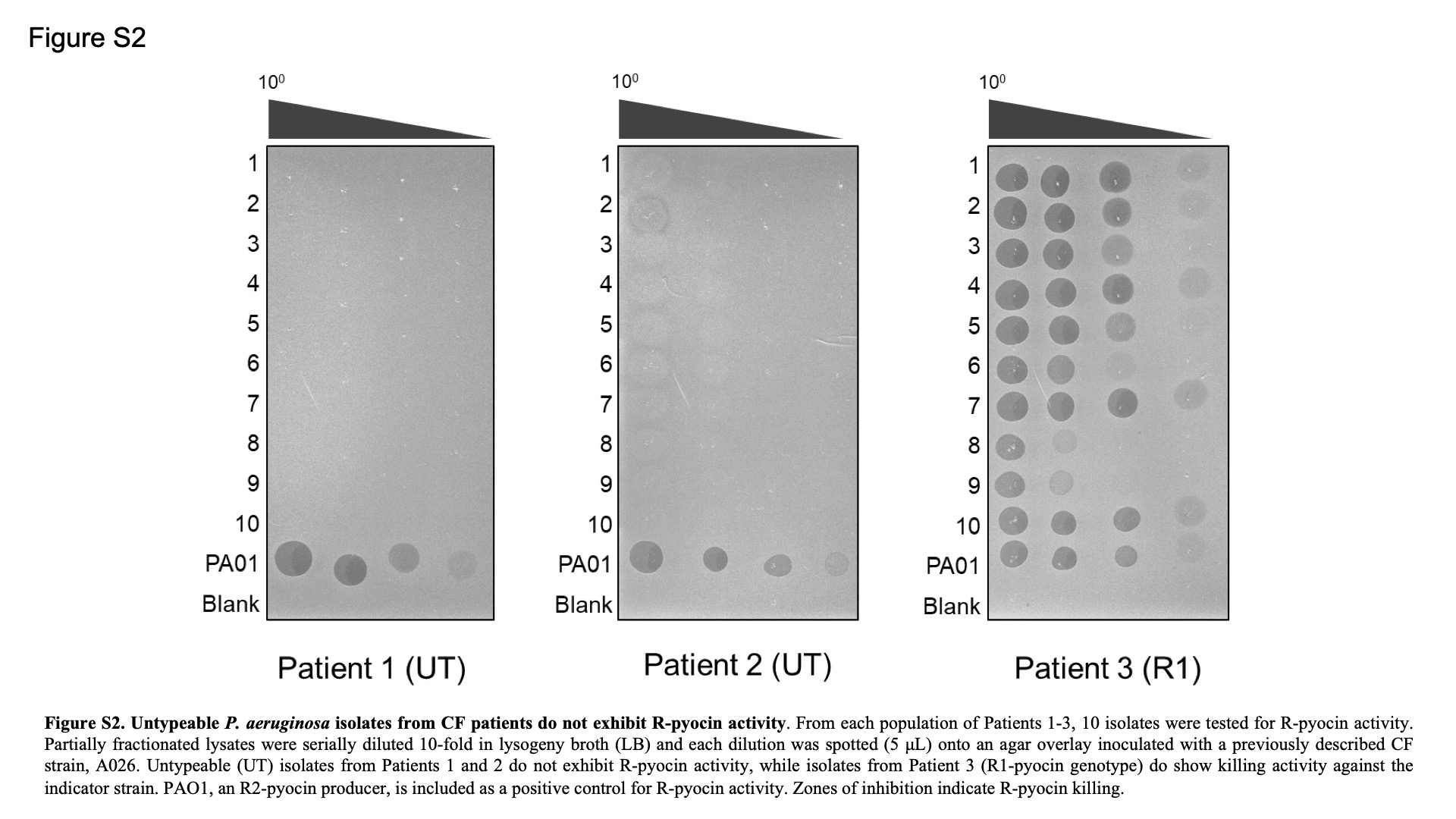

Supplement: FIG S2 [file mbio.00458-21-sf002.jpg]

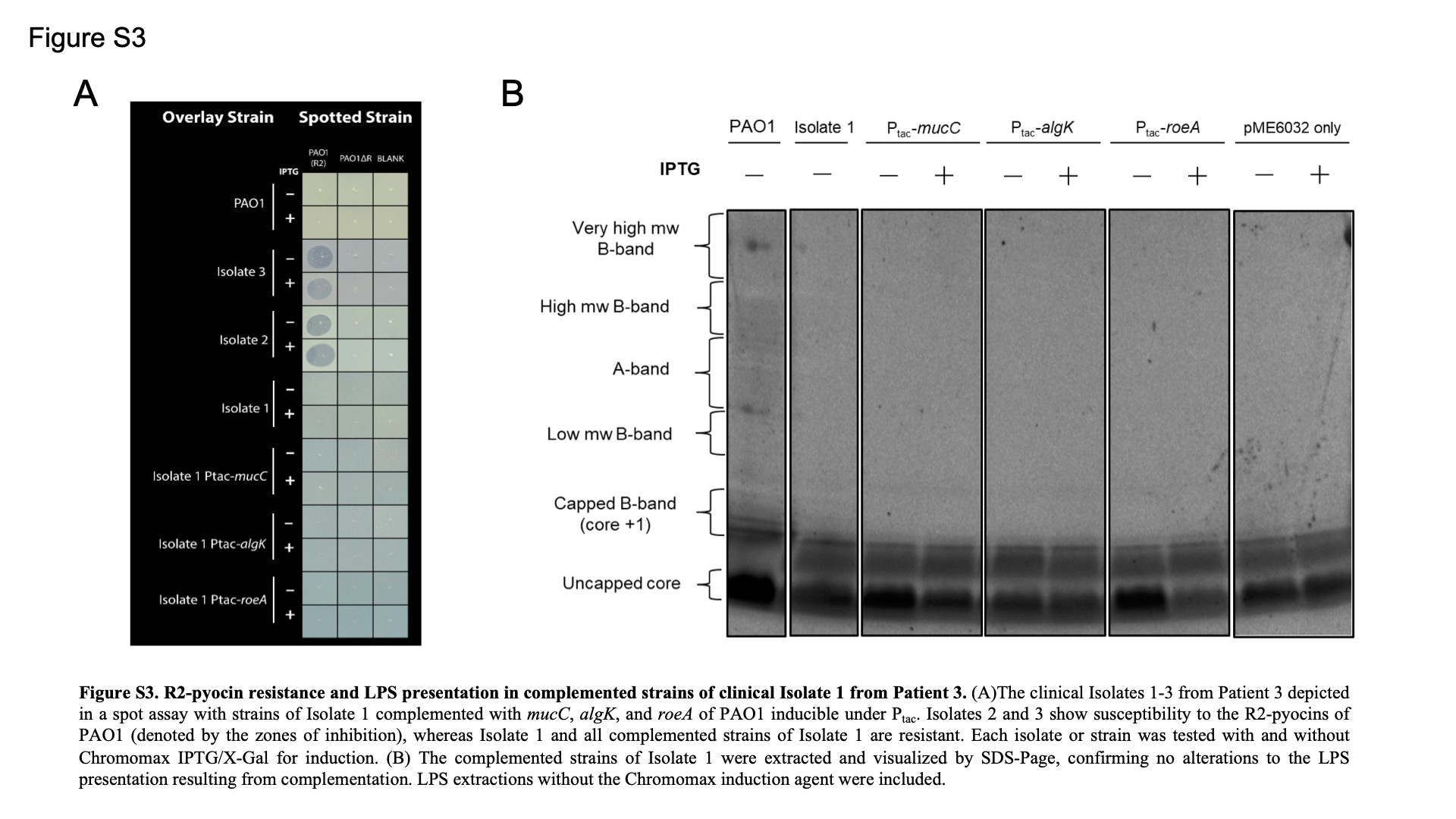

Supplement: FIG S3 [file mbio.00458-21-sf003.jpg]
