# Supplementary material for: Heterogenous Susceptibility to R-Pyocins in Populations of Pseudomonas aeruginosa Sourced from Cystic Fibrosis Lungs
Source: mBio. 2021 May 4;12(3):e00458-21. doi: 10.1128/mBio.00458-21 (PMC8262887; doi:10.1128/mBio.00458-21)
Supplement: TABLE S1 [file mbio.00458-21-st001.docx]

**Table S1.** Primers and Plasmids used in this study.

| **Primer (bp)*** | **Sequence (5’ to 3’)** | **Description** | **Reference or Source** |
| --- | --- | --- | --- |
| R1-Forward (441) | ATGATTTTTTTCCATGCCGCCACG | R-pyocin typing; gives fragment for R1 tail fiber | (1) |
| R1-Reverse | TCAGGGGGTGATGAGCGATTGG | R-pyocin typing; gives fragment for R1 tail fiber | (1) |
| R2-Forward (257) | ATGCCGATGCTTCGATTAC | R-pyocin typing; gives fragment for R2 tail fiber | (1) |
| R2-Reverse | AAACCTCTCGCAAGGAGG | R-pyocin typing; gives fragment for R2 tail fiber | (1) |
| R5-Forward (601) | AATGCACAGGCCGAAAGTGGGG | R-pyocin typing; gives fragment for R5 tail fiber | This study |
| R5-Reverse | TGACATCTGCGAGGGTGACGGT | R-pyocin typing; gives fragment for R5 tail fiber | This study |
| TF-del upstream-Forward (600) | GAGCTCAGGTTACCCGCATGGGTCCTGGATGAAACCCAGG | R-pyocin null mutant generation; To clone PA0620-1 600 bp flanking regions into pDM4 for deletion construct | This study |
| TF-del upstream-Reverse | GTAACGCCGTGGAAGAAACTCATGTCACTGTTTCCAGGGGA | R-pyocin null mutant generation; To clone PA0620-1 600 bp flanking regions into pDM4 for deletion construct | This study |
| TF-del downstream-Forward | CCCCTGGAAACAGTGACATGAGTTTCTTCCACGGCGTTACGGTAACCAAC | R-pyocin null mutant generation; To clone PA0620-1 600 bp flanking regions into pDM4 for deletion construct | This study |
| TF-del downstream-Reverse | CACTAGTGGGGCCCTTCTAGCGAAGCCGGGGCGTTGC | R-pyocin null mutant generation; To clone PA0620-1 600 bp flanking regions into pDM4 for deletion construct | This study |
| *mucC-*Forward (521) | ATCGATGCATGCCATGGTACTCAGGCCGAATCGGACGGCCCGGAAATCAGCG | Complementation; To clone PA0756 *mucC* into pME6032 (under P_tac_) in Isolate1 | This study |
| *mucC-*Reverse | TTCACACAGGAAACAGAATTTCCGGCCCGAGGCCGCCGCC | Complementation; To clone PA0756 *mucC* into pME6032 (under P_tac_) in Isolate1 | This study |
| *algk*-Forward (1495) | ATCGATGCATGCCATGGTACTCATAGGCTTTCTGGCTCTTCTTCGTTGATCG | Complementation; To clone PA3543 *algK* into pME6032 (under P_tac_) in Isolate1 | This study |
| *algK*-Reverse | TTCACACAGGAAACAGAATTCCACCGCTCGCTGACCGGTA | Complementation; To clone PA3543 *algK* into pME6032 (under P_tac_) in Isolate1 | This study |
| *roeA*-Forward | ATCGATGCATGCCATGGTACTTACCGCAGGCTTTCCGCGAGGC | Complementation; To clone PA1107 *roeA* into pME6032 (under P_tac_) in Isolate1 | This study |
| *roeA*-Reverse | TTCACACAGGAAACAGAATTGCCGGATAGGGCGGCCGTCG | Complementation; To clone PA1107 *roeA* into pME6032 (under P_tac_) in Isolate1 | This study |
|  |  |  |  |
| **Plasmids** | **Description** | **Source** |  |
| pDM4 | Suicide vector, Cm^r^ | Nottingham Collection |  |
| pJT95 | pDM4 bearing construct gene with deletion of PA0620 and PA0621; Cm^r^ | This study |  |
| pME6032 | Expression vector, Tc^r^ | Nottingham Collection |  |
| pMM01 | pME6032 containing PA0756 gene under inducible P_tac_ (pME6032::*mucC*), Tc^r^ | This study |  |
| pMM02 | pME6032 containing PA3543 gene under inducible P_tac_ (pME6032::*algK*), Tc^r^ | This study |  |
| pMM03 | pME6032 containing PA1107 gene under inducible P_tac_ (pME6032::*roeA*), Tc^r^ | This study |  |

*The size of the product of each primer pair is given as base pairs in parentheses.
