# Supplementary material for: Heterogenous Susceptibility to R-Pyocins in Populations of Pseudomonas aeruginosa Sourced from Cystic Fibrosis Lungs
Source: mBio. 2021 May 4;12(3):e00458-21. doi: 10.1128/mBio.00458-21 (PMC8262887; doi:10.1128/mBio.00458-21)
Supplement: TEXT S1 [file mbio.00458-21-s0001.docx]

**Text S1**

**Generation of mutants and complemented strains.** Pyocin tail mutants were constructed as follows: 600 bp DNA sequences flanking the open reading frames of PA0620-PA0621 were PCR amplified using Q5 DNA polymerase (New England Biolabs, Ipswich, MA). These sequences were cloned into the SphI-XbaI digested suicide vector pDM4 (8) by Gibson assembly using NEBuilder HiFi Assembly master mix (New England Biolabs), transformed into *Escherichia coli* S17-λpir by electroporation and selected on LB agar plates supplemented with 30 µg/mL chloramphenicol. Cloned inserts were verified by colony PCR and Sanger sequencing (Eurofins Genomics, Louisville, KY). Deletion constructs were introduced into PAO1 and other strains by electroporation (9) and strains carrying single crossover insertions of the deletion constructs were selected on LB agar plates supplemented with 300 µg/mL chloramphenicol. Chloramphenicol resistant colonies were cultured in LB without antibiotic and plated on LB agar plates with 0.25% NaCl and 5% sucrose (10) to select for loss of the deletion construct. Sucrose resistant colonies were screened for chloramphenicol sensitivity to ensure loss of the vector sequence and were assessed for presence of the gene deletion by colony PCR and Sanger sequencing of the PCR product.

Complemented strains of Isolate 1 from Patient 3 were constructed as follows: DNA sequences of each gene from PAO1 Nottingham strain (mucC PA0765; algK PA3543; roeA PA1107) were PCR amplified using Q5 DNA polymerase (New England Biolabs, Ipswich, MA). These sequences were cloned into the EcoRI-KpnI digested expression vector pME6032 (11) to be under inducible control of the tac promoter by Gibson assembly using NEBuilder HiFi Assembly master mix (New England Biolabs), transformed into *Escherichia coli* S17-λpir by electroporation and selected on LB agar plates supplemented with 10 µg/mL tetracycline. Cloned inserts were verified by colony PCR and Sanger sequencing (Eurofins Genomics, Louisville, KY). Expression constructs were introduced into Isolate 1 by electroporation (9) and strains carrying the complementation constructs were selected on LB agar plates supplemented with 200 µg/mL tetracycline.

**R-pyocin typing strains of the IPCD.** The variable region to be used for typing was determined by identifying the query coverage between each pairwise alignment, subtracting the “uncovered” portion from the original base pair length of the query, which suggested approximated 800bp corresponding to the C-terminal end of the tail fiber gene that is most variable to the other types (R1) (Supplementary Data 2). Specifically the 800bp C-terminal sequence of each R-type of tail fiber sequence were used to further R-pyocin type the database. Genomes from the International Pseudomonas Consortium Database (IPCD; BioProject ID 325248) were downloaded from NCBI as nucleotide sequences and made into a “database” to use with blast+ locally (5-6, 8-12). Default BLAST parameters were used to align each R-type sequence with sequences in the IPCD database, to generate tables of IPCD strains for each R-pyocin type (12). Strains were considered to be of a R-pyocin genotype if they covered 99% or greater of the 800bp query sequence, and were determined to be of 96% or higher homology (identity) to the query sequence. A Genbank file of the IPCD was also retrieved from NCBI and used to extract information (source, strain, host, etc) for each strain, to analyze distribution of strains and sources of each R-type (5). Due to the curation and labeling of the strain information in the database, we were not able to precisely distinguish CF strains from strains isolated from other respiratory infections; CF isolates were considered with isolates of other respiratory sources.

**PCR conditions and R-pyocin typing.** Primers for typing R1- and R2-pyocins were designed using the *P. aeruginosa* Genome Database as a reference (<http://www.pseudomonas.com>) (13) in a previous study (1). Primers for typing R5-pyocins were designed using the publicly available genome sequence of strain E429, isolate 15108-1 (NZ_MCME00000000) of the International Pseudomonas Consortium Database (5) ; this strain has been reported as an R5-type pyocin producer by other groups (6). The R5 primers were inputted into the Sequence Manipulation Suite (<https://www.bioinformatics.org/sms2/pcr_products.html>) (14) to verify specificity to R5-pyocin producers and product size. R5-pyocin primers were designed using Benchling (www.benchling.com) (15) and ordered from Eurofins Genomics. R-pyocin typing primer details and product sizes are listed in Table S1. Clinical isolates were R-pyocin-typed with multiplex PCR comprising of R1, R2, and R5 primers, with laboratory strains already available PAO1 (R2), PAK (R1) and TuD199 (R5) used as positive controls for each PCR (1-2, 4-6). The PCR volume of 15 µL contained 3 µL of One *Taq* Standard Reaction Buffer, 0.3 µL of each primer (10 µM) and deoxynucleoside triphosphates (dNTPs [10nM]), 0.075 µL of One *Taq* DNA polymerase and 10.225 µL of nuclease-free deionized water. Template DNA was obtained by using 1 colony per reaction. The PCR conditions used included 94° for 30 s during initial denaturation, followed by 30 cycles of 94°C for 30 s, 58°C for 30 s, 68°C for 30 s, followed by final extension at 68°C for 5 minutes.

**Expression and extraction of R-pyocins.** LB cultures of PAO1 and PAO1ΔR (R-pyocin null mutant) were inoculated at 1:100 from overnight planktonic cultures, and grown to mid-logarithmic growth phase (approximately 3 hours) in 10mL of LB media. Ciprofloxacin was then added to each culture for a final concentration of 0.1µg/mL to induce R-pyocin production (16), and cultures were incubated for a further 3 hours. Chloroform was used to lyse remaining cells and inactivate filamentous prophage (17-19), and lysates were centrifuged at ~3,300 x *g* for 10 minutes. The R-pyocin-containing supernatant was separated and stored at 4°C. R-pyocin lysates were extracted on three separate occasions for biological replication.

**Microtiter plate method for R-pyocin activity.** Susceptibility to R2-pyocins was measured by fold-change of the optical density (OD_600nm_) of each culture treated with R-pyocin-containing lysates, normalized by the optical density of the culture treated with PAOΔR-pyocin mutant lysates after 4 hours of growth. This normalization allows for the consideration of growth heterogeneity and response to any non-R-pyocin particles in the lysates, confirming that the variation in response seen among isolates is R-pyocin-dependent. A standard 96-well plate was used to assess R-pyocin susceptibility of *P. aeruginosa* clinical isolates. Using mid-log phase cultures of the clinical isolates (grown separately), each well of the plate contained LB broth and was inoculated to an adjusted OD_600nm_ of 0.01, before 10µL of R-pyocin lysates were added for a total volume of 200µL in each well. Cultures measured included normal growth cultures of each isolate with no R-pyocin lysates added, cultures with R-pyocin lysates, cultures with R-pyocin null mutant lysates, and cultures with blank media “lysates” added. Using a BioTek Synergy H1, OD_600nm_ was measured for each well every 20 minutes over a total of 16 hours while incubating at 37°C with 200rpm orbital shaking. Growth rates of isolates not treated with R-pyocins were calculated using Growthcurver in R (20-21). Microtiter plate assays for R-pyocin susceptibility were conducted in triplicate. Colony forming units (CFUs) were quantified at t=0 hours and t=4 hours by sampling 10µL of each culture, serially diluting in phosphate buffered saline (PBS), and spotting on LB plates. Colonies were counted after 18 hours of growth at 37°C. CFUs were determined for three biological replicates of each isolate.

**Alginate Isolation and Quantification**. Alginate was isolated as previously described (23). Single colonies of each isolate were used to inoculate 3 mL of LB and grown to mid-logarithmic phase at 37°C, 200 rpm. Using a BioTek Synergy H1, OD_600nm_ was measured and used to inoculate fresh overnight cultures of 20 mL of LB at an OD_600nm_ of 0.01. 10 mL of 0.85% sodium chloride (Fisher Scientific) was combined with 10 mL of overnight culture, vortexed, and centrifuged at ~3,300 x *g* for 30 minutes. Supernatant was removed and combined with 20 mL of 2% cetylpyridinium chloride (Sigma), inverted to precipitate the alginate (10 times), and centrifuged at ~3,300 x *g* for 10 minutes. The supernatant was poured off and discarded, preserving the pellet. 10 mL of 1 M sodium chloride was used to resuspend the alginate pellet and vortexed before incubating at room temperature for 30 minutes. 10 mL of isopropanol (Fisher Scientific) was added to the alginate solution, inverted 10 times, vortexed, and centrifuged at ~3,300 x *g* for 10 minutes. The supernatant was poured off and discarded. The alginate pellet was then resuspended in 10 mL of 0.85% sodium chloride and incubated at 4°C overnight.

Following the incubation of the isolated alginate samples in a borate-carbazole solution at 55°C (22-23), a BioTek Synergy H1 was used to measure the OD_530nm_ . All values were compared to a standard curve generated by diluting laboratory grade alginic acid (Sigma) in 0.85% sodium chloride to concentrations of 50-1000 ug/ml. Alginate production was analyzed and compared for three biological replicates for Isolates 1-3 of Patient 3 by comparing plate reader values to the standard curve generated.

**LPS extraction and characterization.** Overnight bacterial cultures of 5 mL LB were grown at 37°C at 200 rpm shaking. Complemented Isolate 1 strains were grown in 5mL LB supplemented with 200 µg/mL tetracycline to maintain the construct, and 20 µL Chromomax IPTG/X-Gal solution (Fisher Scientific) for induction of gene expression. The overnight cultures were diluted 1:10 with LB for an OD_600nm_ measurement and diluted to make a 1.5 mL suspension of bacteria adjusted to an OD_600nm_ of 0.5 before pelleting by centrifugation at 10,600x *g* for 10 minutes. The cell pellet was resuspended in 200 µL of 1x SDS-buffer. Suspended bacterial cells were boiled for 15 minutes and LPS were prepared by hot aqueous-phenol extraction as previously described (24) but without the addition of DNase I and RNase solutions (as deemed optional in the protocol). Samples were visualized using 15 µL of LPS preparation on a 4%-12% gradient Tris-glycine gel using the Pro-Q Emerald 300 Lipopolysaccharide Gel Stain Kit (ThermoFisher) (24).

**Whole genome sequencing analysis.** Genomic DNA was prepared from overnight cultures using the DNeasy UltraClean Microbial Kit (Qiagen). Library prep and sequencing was performed by the Georgia Institute of Technology Molecular Evolution Core Facility. We obtained 250 bp, paired-end reads sequenced on the Illumina MiSeq platform for an average of at least 60X coverage. Adapters were trimmed following sequencing before receipt for analysis. Reads were checked for quality with FastQC (25), before mapping with default parameters to the PAO1 reference genome (NC_002516) using BowTie2 (version 2.1.0) (26-27). SNV lists between each clinical isolate and PAO1 were generated using Samtools (version 0.1.19) and filtered for a minimum quality score of 20 (28-29). To annotate SNV lists, the same PAO1 reference files used for mapping were built into a database to annotate SNV lists through SnpEff version 4.3t (30). SNV lists were compared across all three isolates for shared SNVs and visualized for comparison with R (version 3.5.1) (21). The full list of SNVs unique to each isolate can be found in Supplementary Data 3. Sam files generated from mapping each isolate to PAO1 were used to visualize coverage with BRIG (version 0.95) (31) and CGView (32). Sequences were compiled into de novo assemblies using SPAdes (33) through KBASE (34) for serotype prediction with PAst 1.0 (35) and strain typing with PubMLST (36).
