# Supplementary material for: Heterogenous Susceptibility to R-Pyocins in Populations of Pseudomonas aeruginosa Sourced from Cystic Fibrosis Lungs
Source: mBio. 2021 May 4;12(3):e00458-21. doi: 10.1128/mBio.00458-21 (PMC8262887; doi:10.1128/mBio.00458-21)
Supplement: TEXT S2 [file mbio.00458-21-s0002.docx]

**Text S2**

1. Oluyombo O, Penfold CN, Diggle SP. 2019. Competition in Biofilms between Cystic Fibrosis Isolates of *Pseudomonas aeruginosa* Is Shaped by R-Pyocins. mBio 10:e01828-18.
2. Holloway BW. 1955. Genetic Recombination in *Pseudomonas aeruginosa*. Microbiology 13:572-581.
3. Takeya K, Amako K. 1966. A rod-shaped Pseudomonas phage. Virology 28:163-5.
4. Pirnay JP, Bilocq F, Pot B, Cornelis P, Zizi M, Van Eldere J, Deschaght P, Vaneechoutte M, Jennes S, Pitt T, De Vos D. 2009. *Pseudomonas aeruginosa* population structure revisited. PLoS One 4:e7740.
5. Freschi L, Jeukens J, Kukavica-Ibrulj I, Boyle B, Dupont M-J, Laroche J, Larose S, Maaroufi H, Fothergill JL, Moore M, Winsor GL, Aaron SD, Barbeau J, Bell SC, Burns JL, Camara M, Cantin A, Charette SJ, Dewar K, Déziel É, Grimwood K, Hancock REW, Harrison JJ, Heeb S, Jelsbak L, Jia B, Kenna DT, Kidd TJ, Klockgether J, Lam JS, Lamont IL, Lewenza S, Loman N, Malouin F, Manos J, McArthur AG, McKeown J, Milot J, Naghra H, Nguyen D, Pereira SK, Perron GG, Pirnay J-P, Rainey PB, Rousseau S, Santos PM, Stephenson A, Taylor V, Turton JF, Waglechner N, et al. 2015. Clinical utilization of genomics data produced by the international *Pseudomonas aeruginosa* consortium. Frontiers in Microbiology 6.
6. Köhler T, Donner V, van Delden C. 2010. Lipopolysaccharide as Shield and Receptor for R-Pyocin-Mediated Killing in *Pseudomonas aeruginosa*. Journal of Bacteriology 192:1921-1928.
7. Simon R, Priefer U, Puhler A. 1983. A broad host range mobilization system for in vivo genetic engineering: transposon mutagenesis in Gram negative bacteria. Nat Biotechnol 1:784-791.
8. Milton DL, O'Toole R, Horstedt P, Wolf-Watz H. 1996. Flagellin A is essential for the virulence of *Vibrio anguillarum*. Journal of Bacteriology 178:1310-1319.
9. Choi K-H, Kumar A, Schweizer HP. 2006. A 10-min method for preparation of highly electrocompetent *Pseudomonas aeruginosa* cells: Application for DNA fragment transfer between chromosomes and plasmid transformation. Journal of Microbiological Methods 64:391-397.
10. Hmelo LR, Borlee BR, Almblad H, Love ME, Randall TE, Tseng BS, Lin C, Irie Y, Storek KM, Yang JJ, Siehnel RJ, Howell PL, Singh PK, Tolker-Nielsen T, Parsek MR, Schweizer HP, Harrison JJ. 2015. Precision-engineering the *Pseudomonas aeruginosa* genome with two-step allelic exchange. Nature Protocols 10:1820-1841.
11. Wilton R, Ahrendt AJ, Shinde S, Sholto-Douglas DJ, Johnson JL, Brennan MB, Kemner KM. 2018. A New Suite of Plasmid Vectors for Fluorescence-Based Imaging of Root Colonizing Pseudomonads. Front Plant Sci. 1;8:2242.
12. Camacho C, Coulouris G, Avagyan V, Ma N, Papadopoulos J, Bealer K, Madden TL. 2009. BLAST+: architecture and applications. BMC Bioinformatics 10:421.
13. Winsor GL, Griffiths EJ, Lo R, Dhillon BK, Shay JA, Brinkman FS. 2016. Enhanced annotations and features for comparing thousands of Pseudomonas genomes in the Pseudomonas genome database. Nucleic Acids Res 44:D646-53.
14. Stothard P. 2000. The sequence manipulation suite: JavaScript programs for analyzing and formatting protein and DNA sequences. Biotechniques 28:1102, 1104.
15. Anonymous. 2019. Benchling [Biology Software].
16. Williams SR, Gebhart D, Martin DW, Scholl D. 2008. Retargeting R-type pyocins to generate novel bactericidal protein complexes. Applied and environmental microbiology 74:3868-3876.
17. Roberts, LM., Dunker AK. 1993. Structural changes accompanying chloroform-induced contraction of the filamentous phage fd. Biochemistry 32: 10479-10488.
18. Griffith J, Manning M, Dunn K. 1981. Filamentous bacteriophage contract into hollow spherical particles upon exposure to a chloroform-water interface. Cell 23:747-753.
19. Olofsson L, Ankarloo J, Andersson PO, Nicholls IA. 2001. Filamentous bacteriophage stability in non-aqueous media. Chemistry & Biology 8:661-671.
20. Sprouffske K, Wagner A. 2016. Growthcurver: an R package for obtaining interpretable metrics from microbial growth curves. BMC Bioinformatics 17:172.
21. Team RC. 2013. R: A language and environment for statistical computing.
22. Knutson, CA, Jeanes A. 1968. A new modification of the carbazole analysis: Application to heteropolysaccharides. Analytical Biochemistry 24: 470-481.
23. Cross, AR, Goldberg JB. 2019. Remodeling of O Antigen in Mucoid *Pseudomonas aeruginosa* via Transcriptional Repression of wzz2. mBio. 10: e02914-02918.
24. Davis MR, Jr., Goldberg JB. 2012. Purification and visualization of lipopolysaccharide from Gram-negative bacteria by hot aqueous-phenol extraction. J Vis Exp doi:10.3791/3916.
25. Andrews S. 2015. FastQC.
26. Langmead B, Salzberg SL. 2012. Fast gapped-read alignment with Bowtie 2. Nat Methods 9:357-9.
27. Langmead B, Wilks C, Antonescu V, Charles R. 2019. Scaling read aligners to hundreds of threads on general-purpose processors. Bioinformatics 35:421-432.
28. Li H, Handsaker B, Wysoker A, Fennell T, Ruan J, Homer N, Marth G, Abecasis G, Durbin R. 2009. The Sequence Alignment/Map format and SAMtools. Bioinformatics 25:2078-9.
29. Li H. 2011. A statistical framework for SNP calling, mutation discovery, association mapping and population genetical parameter estimation from sequencing data. Bioinformatics 27:2987-93.
30. Cingolani P, Platts A, Wang le L, Coon M, Nguyen T, Wang L, Land SJ, Lu X, Ruden DM. 2012. A program for annotating and predicting the effects of single nucleotide polymorphisms, SnpEff: SNPs in the genome of *Drosophila melanogaster* strain w1118; iso-2; iso-3. Fly (Austin) 6:80-92.
31. Alikhan N-F, Petty NK, Ben Zakour NL, Beatson SA. 2011. BLAST Ring Image Generator (BRIG): simple prokaryote genome comparisons. BMC Genomics 12:402.
32. Grant JR, Stothard P. 2008. The CGView Server: a comparative genomics tool for circular genomes. Nucleic acids research 36:W181-W184.
33. Nurk S, Bankevich A, Antipov D, Gurevich A, Korobeynikov A, Lapidus A, Prjibelsky A, Pyshkin A, Sirotkin A, Sirotkin Y, Stepanauskas R, McLean J, Lasken R, Clingenpeel SR, Woyke T, Tesler G, Alekseyev MA, Pevzner PA. Assembling Genomes and Mini-metagenomes from Highly Chimeric Reads, p 158-170. In (ed), Springer Berlin Heidelberg.
34. Arkin AP, Cottingham RW, Henry CS, Harris NL, Stevens RL, Maslov S, Dehal P, Ware D, Perez F, Canon S, Sneddon MW, Henderson ML, Riehl WJ, Murphy-Olson D, Chan SY, Kamimura RT, Kumari S, Drake MM, Brettin TS, Glass EM, Chivian D, Gunter D, Weston DJ, Allen BH, Baumohl J, Best AA, Bowen B, Brenner SE, Bun CC, Chandonia J-M, Chia J-M, Colasanti R, Conrad N, Davis JJ, Davison BH, DeJongh M, Devoid S, Dietrich E, Dubchak I, Edirisinghe JN, Fang G, Faria JP, Frybarger PM, Gerlach W, Gerstein M, Greiner A, Gurtowski J, Haun HL, He F, Jain R, et al. 2018. KBase: The United States Department of Energy Systems Biology Knowledgebase. Nature Biotechnology 36:566-569.
35. Thrane SW, Taylor VL, Lund O, Lam JS, Jelsbak L. 2016. Application of Whole-Genome Sequencing Data for O-Specific Antigen Analysis and In Silico Serotyping of *Pseudomonas aeruginosa* Isolates. Journal of Clinical Microbiology 54:1782-1788.
36. Jolley KA, Bray JE, Maiden MCJ. 2018. Open-access bacterial population genomics: BIGSdb software, the PubMLST.org website and their applications. Wellcome Open Res 3:124.
